# Features of Chaperone Induction by 9-Aminoacridine and Acridine Orange

**DOI:** 10.3390/bios15120800

**Published:** 2025-12-06

**Authors:** Vadim V. Fomin, Svetlana V. Smirnova, Sergey V. Bazhenov, Aminat G. Kurkieva, Nikolay A. Bondarev, Daria M. Egorenkova, Daniil I. Sakharov, Ilya V. Manukhov, Serikbai K. Abilev

**Affiliations:** 1 Moscow Center for Advanced Studies, Moscow 123592, Russia; 2 Vavilov Institute of General Genetics, Russian Academy of Sciences, Moscow 119991, Russia

**Keywords:** lux-biosensor, heat shock, 9-aminoacridine, acridine orange, luminescence

## Abstract

The fluorescent dyes 9-aminoacridine (9-AA) and acridine orange (AO) are known mutagens that induce frameshift mutations in cells by intercalating between DNA bases. However, these chemicals can also affect other cellular components, such as proteins. In this study, we tested the ability of 9-AA and AO to induce heat shock in bacteria using the following methods: lux-biosensors based on *Escherichia coli* cells with the *luxCDABE* genes transcriptionally fused to heat shock-specific inducible promoters, RT-qPCR, and nanoDSF. We demonstrated that acridine dyes not only induce mutagenesis but also cause heat shock in bacterial cells. AO significantly reduced the melting temperature of proteins and strongly activated σ^E^- and σ^32^-dependent promoters, but not P*luxC*, which is activated by elevated temperatures via a different mechanism. In contrast, 9-AA weakly denatured the proteins and induced the σ^E^-dependent promoter; however, it activated the σ^32^-dependent promoters and P*luxC*, supporting the hypothesis that the σ^32^ heat shock response system is activated via hairpin RNA denaturation by 9-AA. The study on the application of lux-biosensors was hampered by the high general toxicity and luminescence shielding effect of AO, and RT-qPCR’s sensitivity was insufficient for detection of the response to 9-AA. Thus, methodologically, it is justified to conduct a comprehensive study of substances that cause heat shock or affect bioluminescence by both RT-qPCR and lux-biosensors.

## 1. Introduction

Acridine is a heterocyclic core consisting of three fused-ring heterocyclic moieties and containing nitrogen in the ring structure, and its derivatives are planar polycyclic aromatic molecules that bind tightly and reversibly to DNA via intercalation, without forming covalent bonds. Acridine compounds are used pharmacologically as antimalarial [1], antiseptic, antifungal [2], and antiviral [3] agents, and their derivatives have been intensively studied as potential anticancer drugs [4]. However, their clinical use is limited or even excluded because of side effects [5]. The most comprehensive summary of studies on the antitumor activity among 97 acridines, including derivatives of 9-aminoacridine, nitroacridine, aniline acridine, quinacridane, and others, is provided in the review [6].

**Figure A1:**
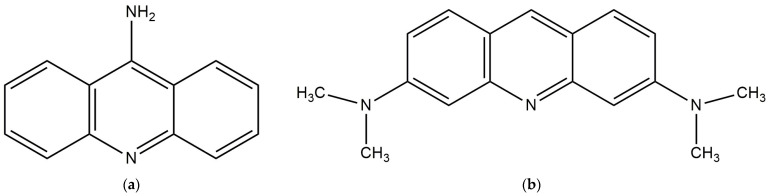
Chemical structures of (**a**) 9-aminoacridine (9-AA) and (**b**) acridine orange (AO).

9-Aminoacridine (9-AA) and acridine orange (AO) (see Appendix A, Figure A1), which are used in scientific research as fluorescent dyes, are mutagens that cause frameshift mutations in bacterial cells through intercalation between DNA bases [7]. Furthermore, 9-AA serves as a basic chemical scaffold for the synthesis of potential anticancer agents [6]. Studies over the past decade have demonstrated that certain 9-AA derivatives can inhibit topoisomerase II (TopoII) by incorporating into DNA and stabilizing the TopoII-DNA cleavable complex [8,9,10]. This stabilization results in the accumulation of double-strand DNA breaks, ultimately triggering cell cycle arrest and apoptosis. For instance, amsacrine has been approved for clinical use in treating leukemia and lymphoma. It is the first topoisomerase II inhibitor—an antitumor derivative of 9-AA acridine [9,11]. In addition, 9-AA can inhibit both the transcription of ribosomal RNA precursors (pre-rRNA) and the processing of already synthesized pre-rRNA, leading to the disruption of ribosome biogenesis [12].

Unlike 9-AA, AO has an acridine core with two dimethylamino side groups. AO is a fluorescent molecule typically excited in the blue spectral region (450–500 nm) and emitting green light (500–600 nm) under highly dilute conditions.

AO is a lysotropic dye that accumulates in acidic organelles, such as lysosomes and/or autophagolysosomes, and, upon photo- or radiodynamic activation, induces lysosome-mediated cell death. When stained with AO, the cytoplasm and nucleoli typically fluoresce green, whereas acidic organelles exhibit bright red or orange-red fluorescence under blue light excitation. These properties of AO have led to the development of photodynamic therapy for sarcomas [13,14]. AO-RDT has demonstrated selective toxicity against carcinoma cells and effectively suppresses both tumor growth in bone and tumor-associated osteolysis [15]. Furthermore, photodynamic activation of AO can be used in human bladder cancer (BC) [16].

The ability of chaperones to provide cancer cells with resistance to many DNA-targeted anticancer drugs was described a long time ago [17]. Thus, the chaperone HSP70, along with other chaperones such as HSP27 and HSP90, is involved in supporting the DNA repair process in tumor cells, which increases the resistance of these cells to cytostatics [18], and chaperone inhibitors increase the effectiveness of radiation therapy [19]. For bacteria, the statement about the protective properties of the heat shock regulon genes against radiation, UV, and DNA-damaging chemicals is also true [20,21]. In this regard, the aim of this study was to determine the ability of 9-AA and AO to induce heat shock, which leads to an increase in the concentration of chaperones in the cell.

For these purposes in this study, we used bacterial lux-biosensors, containing the reporter *luxCDABE*-genes of *Photorhabdus luminescens* under the control of various heat shock-regulated promoters: P*grpE*, P*ibpA*, and P*degP* from *E. coli*, and P*luxC* from *P. luminescens*. The *grpE* gene encodes an ATPase within the DnaKJ/GrpE chaperone [22], and the *ibpA* gene encodes the IbpA co-chaperone [23]. The promoters of both genes are transcribed in a σ^32^-dependent manner. The *degP* gene, located within the σ^E^ regulon, encodes a periplasmic protease [24]. An expression of the *P. luminescens lux*-operon is enhanced in response to heat shock and, if it is cloned in *E. coli* cells, this occurs independently of σ^32^ and σ^E^ [25]. Used biosensors, sensitive to the occurrence of heat shock in *E. coli* cells, respond with an increase in bioluminescence [26,27,28]. The *E. coli* biosensors pSoxS-lux [29] and pDlac [25], which are insensitive to heat shock, were used as a control.

## 2. Materials and Methods

### 2.1. Bacterial Strains, Plasmids, and Cultivation Conditions

*E. coli* K12 MG1655 (obtained from VKPM) was subjected to transformation with biosensor plasmids (Table 1).

LB (Luria–Bertani) medium was used for the incubation and cultivation of bacterial cultures, and 1.5% agar was added to obtain the solid medium. The media contained ampicillin at a concentration of 100 μg/mL.

### 2.2. Chemical Compounds

All chemicals were of analytical grade. 9-AA and AO were purchased from PanEco (Moscow, Russia), and paraquat (methyl viologen) was purchased from Sigma Aldrich (St. Louis, MO, USA). The chemical structures of 9-AA and AO are provided in Appendix A (Figure A1).

### 2.3. Luminescent Response Measurements

The experiments were conducted as follows: 2 mL of a 20 h overnight culture was added to 20 mL of fresh LB broth and incubated for two hours at 30 °C, after which the aliquots were poured into 96-well plates. The tested chemical compounds or water as a negative control were added to the culture in various final concentrations (“0” for water), provided in each experiment, in a 9:1 volume ratio of cell suspension to solution. Then bacterial luminescence dynamics were read by microplate readers: StatFax 4400 (Awareness Technology Inc., Palm City, FL, USA) or Synergy H4 (BioTek, Winooski, VT, USA).

### 2.4. Luminescence Shielding Test

*E. coli* pXen7 (bacterial luciferase—LuxAB) and *E. coli* pLR3 (firefly luciferase—Luc) were incubated as described previously. At the end of incubation, the cell suspensions were divided into 0.18 mL portions in microcentrifuge tubes in two variants: the cell suspension added into a 1.5 mL tube for direct mixing with 9-AA or AO at final concentrations of 0.5, 5, and 50 mM (“in” variant), and the culture introduced into a 0.5 mL tube inserted into a larger 1.5 mL tube without mixing with 9-AA or AO, which are added externally to the 1.5 mL tube at the same concentrations (“out” variant) (Figure S1). Then, luminescent signal measurements were performed before and immediately after introducing the chemical into the specified tube, according to the “in” or “out” variants. To measure the luminescence of *E. coli* pLR3, 1 μg of luciferin was added to the culture to initiate a luminescence reaction. Measurements were performed by a Biotox-7BM luminometer (BioPhysTech, Moscow, Russia). The standard deviation was calculated based on 3 replicates.

### 2.5. RNA Isolation and RT-qPCR

After incubating *E. coli* MG1655 cell suspensions with the tested substances for 120 min, the biomass was dissolved in intactRNA fixative (Eurogen, Moscow, Russia). Afterward, total RNA was isolated using the RNA Solo kit (Eurogen, Moscow, Russia) according to the manufacturer’s instructions. The concentration and quality of the isolated RNA were assessed by a NanoPhotometer^®^ P 330 spectrophotometer (Implen, Munich, Germany).

RT-qPCR on the isolated RNA template was performed using the OneTube RT-qPCR SYBR kit (Eurogen, Moscow, Russia) reagents in a Rotor-Gene Q thermocycler (QIAGEN, Hilden, Germany) according to the manufacturer’s instructions but with a final reverse transcriptase concentration of 0.25X. The *grpE* and *ibpA* genes were amplified by primer pairs CCCCGGAAGAAATTATCATGGATCAG/TTCGGGTCCAGTGGGACGTTAG and ATGCGTAACTTTGATTTATCCCCGCTTT/TTAGTTGATTTCGATACGGCGCGGTTTTTT, respectively. The *16S* rRNA gene was amplified with the following primer pair CGTGCCAGCAGCCGCGGTAATA/GGCCCCCGTCAATTCATTTGAGT. Amplification mode: 50 °C for 15 min, 95 °C for 1 min, 45 cycles: 95 °C for 15 s; 57 °C for 20 s; 72 °C for 25 s. Melting curves: 50 °C for 1 min, from 50 °C to 94 °C in increments of 1 °C–5 s.

Changes in the mRNA levels of the *grpE* and *ibpA* genes, normalized to the *16S* rRNA across all samples, were quantified using the ΔΔ*C*_T_ method [34] according to the following formula:

(1) $$\Delta \Delta C_{T}\;=\;(C_{T,{\mathrm{grpE}/\mathrm{ibpA}\;}_{9\text{-AA/AO}}}\;-\;C_{T,{16S\;}_{9\text{-AA/AO}}})-(C_{T,{\mathrm{grpE}/\mathrm{ibpA}}_{H_{2}O}}\;-\;C_{T,{16S}_{H_{2}O}})$$

where *C*_T_ is the threshold cycle of the amplification curve of the corresponding sample. Changes in mRNA levels induced by 9-AA or AO were calculated relative to water (H_2_O) control.

### 2.6. Melting Point of the Proteins

The melting point of the globule was determined using a prometheus^tm^ Panta (NanoTemper Technologies GmbH, Munich, Germany) by the nanoDSF method. Purified luciferase or L-methionine γ-lyase (MGL) [35] in PBS pH 7.2 buffer with a protein concentration of approximately 0.2 mg/mL was heated in a temperature range from 25 to 90 °C at a rate of 1 °C/min. Fluorescence was measured at 350 and 330 nm with excitation at 280 nm throughout the experiment. The measurements were carried out in 3 technical replicates. The first derivative of the 350/330 ratio was plotted on the graphs. The melting point was determined from the peak on the derivative plot.

## 3. Results

### 3.1. Activation of Luminescence by 9-AA and AO

**Figure 1:**
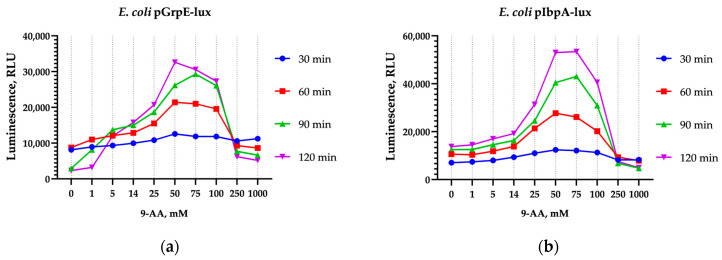
Luminescence induction of (**a**) *E. coli* pGrpE-lux and (**b**) *E. coli* pIbpA-lux incubated with 9-AA in a concentration- and time-dependent manner.

The luminescence of *E. coli* pGrpE-lux and *E. coli* pIbpA-lux cultures increased with the addition of 9-AA at final concentrations of 50 and 75 mM by ~5 times at 90 and 120 min of exposure (Figure 1).

**Figure 2:**
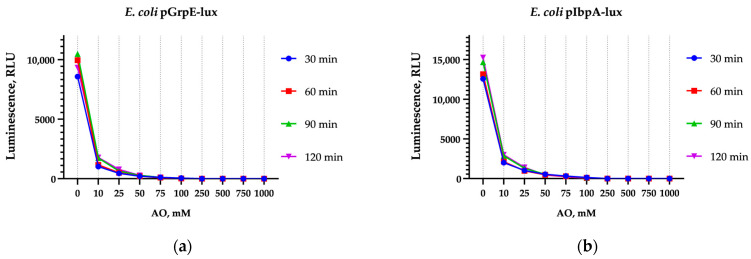
Luminescence inhibition of (**a**) *E. coli* pGrpE-lux and (**b**) *E. coli* pIbpA-lux incubated with AO in a concentration- and time-dependent manner.

In contrast, AO, which differs from 9-AA by the presence of two dimethylamino groups and the absence of an amino group (Figure A1), completely suppressed the luminescence of both biosensors at concentrations of 50 mM and above (Figure 2).

**Figure 3:**
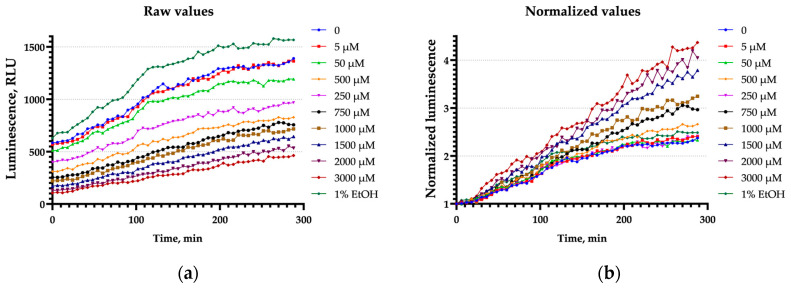
Kinetic curves of *E. coli* pGrpE-lux luminescence supplemented with AO at indicated concentrations and with EtOH as positive control. (**a**) Raw luminescence values obtained from experiments. (**b**) Luminescence values normalized to first data point of each curve.

The data in Figure 2 may indicate a high AO toxicity to cells or the shielding of bioluminescence by effective photon capture. To further investigate the potential for heat shock activation by AO, the kinetic luminescence curves of *E. coli* pGrpE-lux were measured at lower concentrations to minimize shielding and toxicity (Figure 3a). If the decrease in luminescence is primarily due to shielding, then it depends on the AO concentration, appears instantaneously, and is independent of time. To account for possible shielding, the curves were normalized to the first data point after AO addition (Figure 3b).

A decrease in cellular luminescence is observed starting at 50 µM and becomes more pronounced with increasing AO concentration in the medium (Figure 3a). However, the normalized luminescence curves (Figure 3b) demonstrate that AO induces heat shock in a concentration-dependent manner, with a detectable response starting from approximately 500 µM.

### 3.2. Toxicity of AO and 9-AA to E. coli

To test the potential of AO to suppress the *lux*-operon function, we used the *E. coli* pSoxS-lux biosensor, which responds to superoxide anion radical generation induced by paraquat (PQ) [27,36]. Table 2 presents the experimental results, which show that PQ at a concentration of 0.4 mM induces luminescence of the *E. coli* pSoxS-lux biosensor and that the luminescence intensity depends on the exposure time. AO at a concentration of 5 mM reduced the PQ-induced bacterial luminescence, while at 50 mM, the luminescence was completely neutralized. Thus, we demonstrated that AO suppresses *lux*-operon function and decreases luminescence intensity independently of the selected promoter.

To determine the reason for the drop in biosensor luminescence intensity caused by AO, comparative experiments were conducted to evaluate the correlation between the survival of *E. coli* pGrpE-lux bacteria and their luminescence after 60 min of exposure to 9-AA or AO. For this purpose, firstly, bacterial luminescence was measured after incubation, and aliquots from the plate wells were serially diluted to determine the number of colony-forming units (CFU) (Table 3).

Cell titers are reduced by approximately 25-fold and 2-fold due to the toxicity of AO and 9-AA, respectively, at a concentration of 50 mM (Table 3). Despite a slight decrease in bacterial viability at 50 mM 9-AA, an increase in luminescence intensity is observed. Calculation of the specific luminescence of the bacteria—the luminescence values normalized to the number of viable cells—showed that the luminescence level of the *E. coli* pGrpE-lux biosensor decreased approximately 6-fold under exposure to 5 mM AO compared to the control. At the same time, 9-AA and AO at this concentration do not practically reduce bacterial viability. This effect may be associated with the denaturation of the reporter protein, luciferase, and/or luminescence shielding.

### 3.3. Bioluminescence Shielding

**Figure 4:**
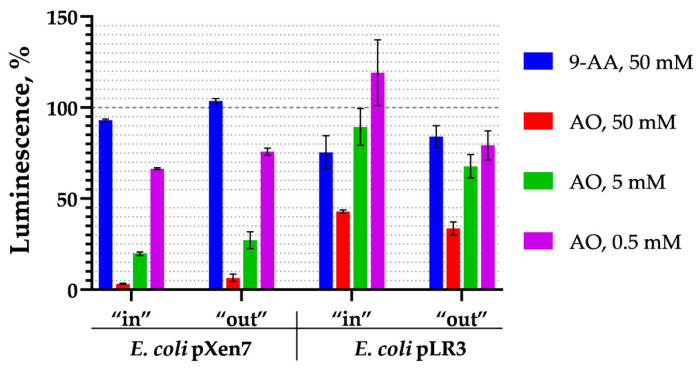
The *E. coli* pXen7 and *E. coli* pLR3 luminescence percentages measured 5 s after the injection of 9-AA or AO according to the “in” and “out” variants. The luminescence values measured prior to the chemical addition were designated as 100%.

To assess bioluminescence shielding by 9-AA and AO, aliquots of *E. coli* pXen7 and *E. coli* pLR3 cell suspensions were distributed into 1.5 and 0.5 mL tubes in two variants, designated as “in” and “out” according to Section 2.4. Thus, when the suspension is in a 0.5 mL tube and the chemical compounds are located outside, between the cell suspension and the detector, only the ability of the chemicals to shield the luminescence signal is taken into account. When 9-AA and AO are directly mixed with the suspensions in a 1.5 mL tube, the effect of toxicity is also considered. The signal is measured for 15 s before and after the addition of the substances (Figures S2 and S3). The percentages of luminescence 5 s after the addition of 9-AA or AO are provided in Figure 4; the luminescence values recorded before the addition moment are set as 100%.

The measured luminescence intensity decreases with increasing AO concentration (Figure 4). The decrease in the “out” variant demonstrates the AO solution’s shielding effect, i.e., the absorption of light quanta. LuxAB luminescence is absorbed more efficiently than Luc due to the AO absorption spectrum: the AO absorption peak is located around 490 nm and is close to the LuxAB emission peak at 485 nm, whereas Luc emits at approximately 565 nm (Figure S4). In addition, the decrease in *E. coli* pXen7 luminescence is greater in the “in” variant than in the “out”, probably because of AO’s toxic and denaturing properties. In the case of 9-AA, no shielding effect is detected. Only a slight decrease in luminescence is observed.

### 3.4. Denaturing Properties of 9-AA and AO

**Figure 5:**
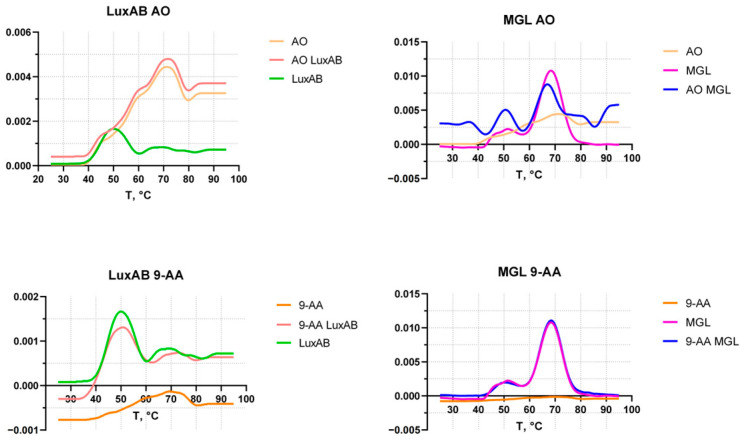
The temperature-dependent first derivative of the melting curves of LuxAB and MGL, with and without the addition of 50 mM 9-AA or AO. Legend: 9-AA and AO—substances without protein; LuxAB and MGL—proteins without substances; 9-AA/AO LuxAB/MGL—mix of both.

Luciferase denaturation as a potential reason for reduced bioluminescence has been investigated. Using the nanoDSF method, the melting temperatures of the globule of the purified LuxAB from *P. luminescens* [26] and of control enzyme MGL were obtained with and without the addition of AO or 9-AA (Figure 5). A concentration of 50 mM AO or 9-AA was used as the critical concentration at which heat shock activation occurs without acute toxicity.

Based on the data presented in Figure 5, 9-AA has a minimal effect on the melting temperature of LuxAB and no detectable effect on the control protein MGL. The melting temperature of the bacterial luciferase is 50.5 °C—the main peak—and 69 °C—the melting peak, apparently, of the globule core. For MGL, the main peak is 68.4 °C, with an additional peak at 51.7 °C, which presumably corresponds to tetramer disintegration. The addition of AO leads to the disappearance of the luciferase melting peaks, indicating protein denaturation before heating, followed by the accumulation of aggregates in solution. In the case of control protein MGL, characterized by a higher melting temperature, the addition of AO leads to a decrease in the temperature of the main peak by 1.6 °C (shifted to 66.8 °C) and a change in the ratio of the main and additional peaks in favor of the additional peak.

### 3.5. mRNA Level of grpE and ibpA Genes

**Figure 6:**
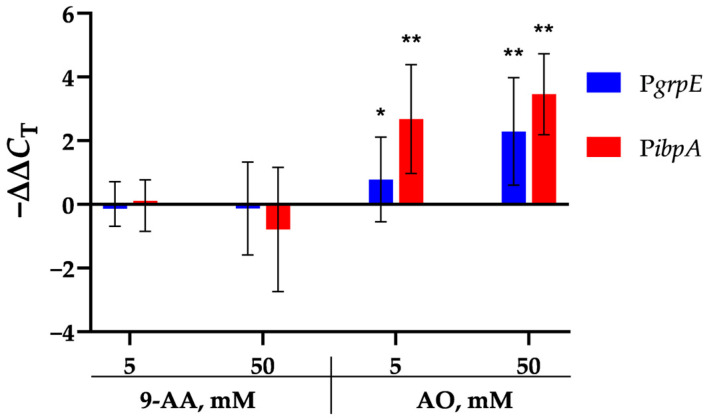
−ΔΔ*C*_T_ values of the *grpE* and *ibpA* genes of *E. coli* MG1655 after 120 min of exposure to 9-AA or AO at the indicated concentrations. The values in the diagram are mean ± SD (*n* = 12 for *ibpA* and 15 for *grpE*). The statistical significance of the changes in mRNA levels was assessed with one-sample *t*-test (H_0_—there is no induction, ΔΔ*C*_T_ is 0): *—*p* < 0.05, **—*p* < 0.01.

The ability of 9-AA and AO to activate transcription of the *grpE* and *ibpA* genes was tested using RT-qPCR, 16S rRNA gene was used for reference. Changes in the mRNA level of *E. coli* MG1655 were assessed by calculating −ΔΔ*C*_T_ after 120 min of exposure to 9-AA or AO (Figure 6).

According to the data in Figure 6, the mRNA levels of the *grpE* and *ibpA* genes remain unchanged after exposure to 9-AA. In contrast, exposure to AO increases the mRNA levels of the *grpE* and *ibpA* genes in a concentration-dependent manner.

### 3.6. Activation of Various Heat Shock Promoters

**Figure 7:**
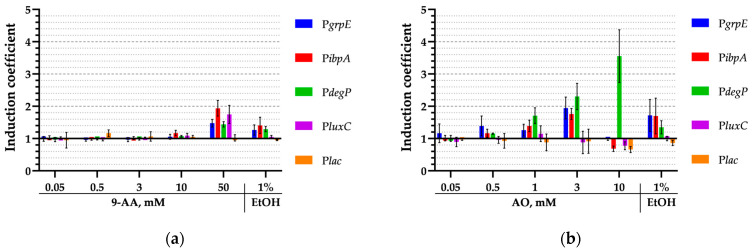
Induction coefficients of biosensors containing heat shock promoters P*grpE*, P*ibpA*, P*degP*, or P*luxC* after 3 h exposure to (**a**) 9-AA or (**b**) AO at various concentrations. Ethanol and P*lac* were used as positive and negative controls, respectively. Standard deviation is calculated based on 3 replicates.

*E. coli* cells have two parallel systems associated with the σ^32^ and σ^E^ regulons that induce the heat shock response. To test the activation of these regulons, biosensors with the following inducible promoters were used: P*grpE* and P*ibpA*, activated by σ^32^; P*degP*, activated by σ^E^; and P*luxC*, from *P. luminescens*, activated in response to heat but not to ethanol, and this activation is independent of the σ^32^ and σ^E^ regulons. The induction coefficients of biosensors with these promoters upon the addition of 9-AA and AO at various concentrations are shown in Figure 7. The induction coefficients were calculated as the ratio of the luminescence value in a test sample to that in a water control sample at the same time point. To account for the AO-mediated decrease in luminescence, the raw luminescence values were normalized to the first data point (Figure 3b) prior to calculating the induction coefficients. The data was obtained after a 3 h exposure to the substances. A cell culture of *E. coli* pDlac, which contains the *lux*-operon under the control of the P*lac* promoter, was used as a negative control.

All tested heat shock promoters, P*grpE*, P*ibpA*, and P*degP*, shown in Figure 7, were activated by 9-AA and AO regardless of whether they belonged to the σ^32^ or the σ^E^ regulons. However, it should be noted that the P*degP* promoter has certain specificity: it is most efficiently induced by AO and much less efficiently by 9-AA, only at the highest concentration of 50 mM.

## 4. Discussion

In this study, we investigated the ability of 9-AA and AO to induce heat shock in *E. coli* cells. The acridine dyes 9-AA and AO are known to intercalate between DNA bases, which often leads to a shift in DNA polymerase and, consequently, to frameshift mutations [37]. The appearance of the acridine dyes in the incubation medium leads to an SOS response in *E. coli* cells caused by replication fork stalling [38]. Here, we tested the possibility that the intercalation ability of acridine dyes could lead to heat shock in bacteria. This hypothesis arose from data on the binding of acridine dyes to proteins, as demonstrated with hemoglobin [39].

In our study, the induction of heat shock-specific biosensors *E. coli* pGrpE-lux and *E. coli* IbpA-lux caused by 9-AA and AO has been shown (Figure 1, Figure 3, and Figure 7). In the case of AO, measurements with biosensors were hampered by a significant decrease in their luminescence (Figure 2, Table 2 and Table 3). The sharp decrease in luminescence upon the addition of AO was independent of the regulatory elements used in the biosensor, which was confirmed with the use of the *E. coli* pSoxS-lux and *E. coli* pDlac biosensors, since the P*soxS* and P*lac* promoters do not belong to any heat shock regulon. In part, this effect is due to shielding: AO efficiently absorbs light from biosensor cells (Figures S2 and S3) because of the coincidence of the maximum luminescence spectrum at 490 nm and the absorption spectrum of AO, which has a close maximum at 495 nm (Figure S4). Since shielding depends on the AO concentration and is independent of time, its effect can be eliminated from the analysis by normalizing the biosensor signal to the first point after the addition of AO (Figure 3). Another mechanism responsible for the significant drop in luminescence caused by AO was the denaturation of the reporter proteins, as confirmed by the nanoDSF melting temperature measurements of the protein globules. AO decreases the melting temperatures of both the bacterial luciferase and the more thermostable MGL protein, leading to the formation of partially denatured proteins in the sample (Figure 5). At high concentrations of AO (>5 mM), the contribution of luciferase denaturation to the observed signal drop from biosensors increases significantly, which leads to a decrease in the biosensor response potential mediated by the synthesis of new luciferase, since it is also susceptible to denaturation.

RT-qPCR studies on the wild-type *E. coli* strain MG1655 did not show activation of mRNA synthesis of the *grpE* and *ibpA* genes upon 9-AA addition. In contrast, AO activates transcription of these genes strongly enough for detection: mRNA level is increased by 1.5- to 3-fold (Figure 6). This creates a contradiction between the results of heat shock studies using both the RT-qPCR technique and lux-biosensors.

The paradox can be explained by the different mechanisms of σ^32^ activation: via the DnaKJ/ClpXP degradation pathway and via a RNA thermometer [40]. We showed that 9-AA does not change the thermostability of proteins in vitro (Figure 5). We propose that 9-AA increases σ^32^ synthesis by intercalating the σ^32^ mRNA palindrome, which closes the ribosome-binding site. Consequently, σ^32^ expression rises, activating the P*grpE* and P*ibpA* promoters. The proposed induction mechanism applies to P*luxC*, which is independent of heat shock regulons and, presumably, has a secondary RNA structure-mediated activation mechanism [25]. Methodologically, this relatively weak effect is poorly detected by RT-qPCR (Figure 6), but is visible to highly sensitive lux-biosensors (Figure 7). The opposite situation occurs with AO, which can effectively denature a protein globule, leading to massive protein denaturation and σ^32^ activation via the DnaKJ/ClpXP system in addition to an RNA thermometer mechanism. This effect is more pronounced and is visible using RT-qPCR, (Figure 6) because the detection of a reliable response of lux-biosensors requires a long exposure time, data processing (Figure 3b), and a selected AO concentration, since the decrease in luminescence at 5 mM reaches approximately 75% (Figure 4) and this decrease is no longer compensated by an increase in the luminescent signal in response to heat shock with an evident σ^32^ induction observed at AO concentration of 3 mM but not 10 mM for P*grpE* and P*ibpA* (Figure 7).

**Figure 8:**
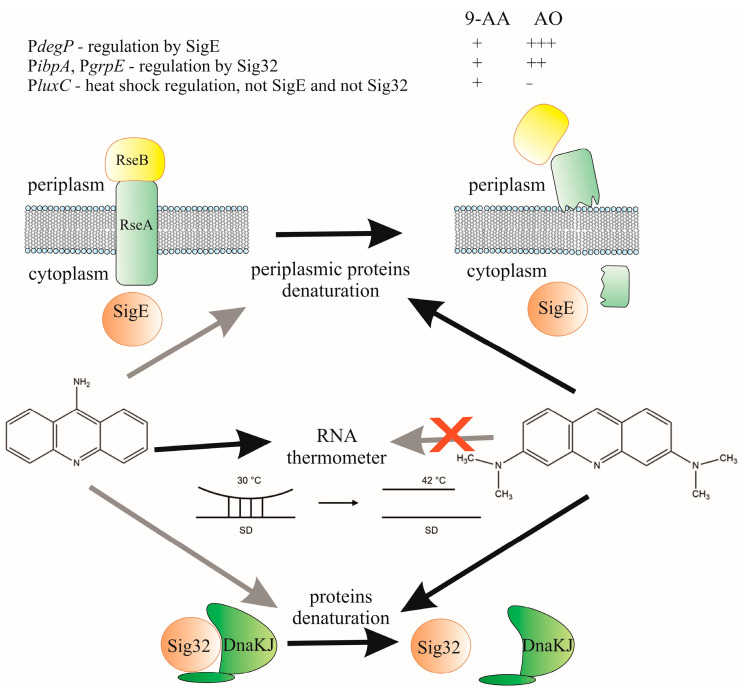
Scheme of heat shock activation by 9-AA and AO. Arrows indicate the effects of substances on the activation of various heat shock mechanisms. Black arrows indicate a strong effect, gray arrows indicate a weak effect, and a crossed-out arrow indicates no effect.

The differences between AO and 9-AA in activating various mechanisms regulating the heat shock response are summarized in Figure 8. Both substances bind DNA (as known from previous publications) and appear to be capable of denaturing proteins. However, this occurs with different efficiencies and specificities. AO demonstrated the ability to significantly reduce protein melting temperatures and induce strong activation of the σ^E^- and σ^32^-dependent promoters, but not P*luxC*, which is activated by elevated temperatures via a different mechanism [25]. 9-AA, in contrast, weakly denatures proteins (low induction of the σ^E^-dependent promoter) but can activate P*luxC*, supporting the hypothesis that the σ^32^ heat shock response system is activated via hairpin RNA denaturation. Knowledge of the ability of acridine dyes to induce thermal stress, specifically disrupt protein structure, can be used to develop a technology for using these dyes in combination with radiation therapy for cancer. However, it should be remembered that the induction of chaperones during thermal stress can reduce the effectiveness of chemotherapy and radiotherapy, so the effect of acridines can be enhanced by adding chaperone inhibitors.

## 5. Conclusions

The obtained data demonstrate the ability of 9-AA and AO to induce heat shock at millimolar concentrations. Also, our study demonstrated the practicability of combining methods for determining promoter activation using both RT-qPCR and lux-biosensors when studying the ability of various drugs and toxicants to induce heat shock in cells.

## Figures and Tables

**Table 1:** Plasmids presented in the study.

Plasmid	Description	Source
pXen7	pUC18 plasmid containing a cloned *lux*-operon from *P. luminescens* ZM1, controlled by its own promoter, Ap^r^	[30]
pGrpE-lux	pDEW201 [31] vector containing a cloned P*grpE* promoter from *E. coli*, transcriptionally fused with the *luxCDABE* genes from *P. luminescens*. Ap^r^	[32]
pSoxS-lux	The same as pGrpE-lux but the P*soxS* promoter is inserted	
pRpoE-lux	The same as pGrpE-lux but the P*degP* promoter is inserted	
pIbpA-lux	The same as pGrpE-lux but the P*ibpA* promoter is inserted	[27]
pDlac	The same as pGrpE-lux but the P*lac* promoter is inserted	[25]
pLR3	pLR derivative containing the *luc* gene from *L. mingrelica*, controlled by P*luxI* promoter from *A. fischeri*, Ap^r^	[33]

**Table 2:** Suppression of *E. coli* pSoxS-lux PQ-induced luminescence values by AO.

Substance, mM	Exposure Time, min			
	15	30	45	60
H_2_O	6874 ± 192	5985 ± 95	4958 ± 103	6028 + 87
PQ, 0.4	6028 ± 87	15,219 ± 986	19,300 ± 1405	30,716 ± 2170
AO, 5	79 ± 10	37± 8	29 ± 7	59 ± 11
AO, 50	0	0	0	0
PQ + AO, 5	85 ± 1	135 ± 20	220 ± 35	436 ± 76
PQ + AO, 50	0	0	0	0

**Table 3:** CFU and luminescence intensity values for *E. coli* pGrpE-lux aliquots from plate well after 60 min exposure to 9-AA or AO.

Substance, mM	CFU, 10^7^	Luminescence, RLU	
		Per well, 10^2^	Per well/CFU, 10^6^
H_2_O	10 ± 5	79	64
9-AA, 5	7 ± 2	90	131
9-AA, 50	5 ± 3	159	233
AO, 5	9 ± 5	8	12
AO, 50	0.4 ± 0.2	0	0

## Data Availability

The data are contained within the article.

## Supplementary Materials

The following supporting information can be downloaded at: https://www.mdpi.com/article/10.3390/bios15120800/s1, Figure S1: (A) Two sample preparation methods for *E. coli* pXen7 and *E. coli* pLR3. Cell suspensions are mixed with 9-AA or AO in a 1.5 mL test tube, which corresponds to the “in” variant (left). Cell suspensions in a small 0.5 mL test tube, which is inserted into a 1.5 mL test tube supplemented with 9-AA or AO (right) according to the “out” variant. Examples are shown before and after the addition of AO. (B) Luminescence measurements for 15 s of *E. coli* pXen7 suspensions prepared according to the “in” and “out” methods, but without the addition of the test substances. The difference in luminescence between the two samples is 1%. Figure S2: Percentage luminescence values obtained according to the method described in the main text of the article for *E. coli* pXen7 (top, LuxAB) and *E. coli* pLR3 (bottom, Luc) according to the “out” (left) and “in” (right) variants, depending on the final AO concentrations: 0.5 (blue), 5 (red), and 50 (green) mM. The data were obtained for 15 s before (dashed line) and 15 s after AO supplementation. The luminescence values at the moment before AO addition are taken as 100% (dash-dotted line). The proportions of luminescence immediately after AO addition are indicated on the graphs by dotted lines; the corresponding numerical values are located near their intersection with the ordinate axis. Figure S3: Percentage luminescence values obtained according to the method described in the main text of the article for *E. coli* pXen7 (left, LuxAB) and *E. coli* pLR3 (right, Luc) according to the “out” (red) and “in” (blue) variants at a 9-AA concentration of 50 mM. The data were obtained for 15 s before (dashed line) and 15 s after 9-AA supplementation. The luminescence values at the moment before 9-AA addition are taken as 100% (dash-dotted line). The proportions of luminescence immediately after 9-AA addition are indicated on the graphs by dotted lines; the corresponding numerical values are located near their intersection with the ordinate axis. Figure S4: Normalized spectra: AO absorption [41], bacterial luciferase (LuxAB) emission [42], and firefly luciferase (Luc) emission [43].
