# Supplementary material for: Features of Chaperone Induction by 9-Aminoacridine and Acridine Orange
Source: Biosensors (Basel). 2025 Dec 6;15(12):800. doi: 10.3390/bios15120800 (PMC12730382; doi:10.3390/bios15120800)
Supplement: Supplementary file 1 [file biosensors-15-00800-s001.zip › biosensors-3993847-supplementary.pdf]

biosensors

MDPI

Supplementary Materials

Features of Chaperone Induction by 9-Aminoacridine and Acridine Orange

Vadim V. Fomin [1], Svetlana V. Smirnova [2], Sergey V. Bazhenov [1], Aminat G. Kurkieva [2], Nikolay A. Bondarev [1], Daria M. Egorenkova [1], Daniil I. Sakharov [1], Ilya V. Manukhov [1,*] and Serikbai K. Abilev [2]

[1] Moscow Center for Advanced Studies, Moscow 123592, Russia
[2] Vavilov Institute of General Genetics, Russian Academy of Sciences, Moscow, 119991, Russia

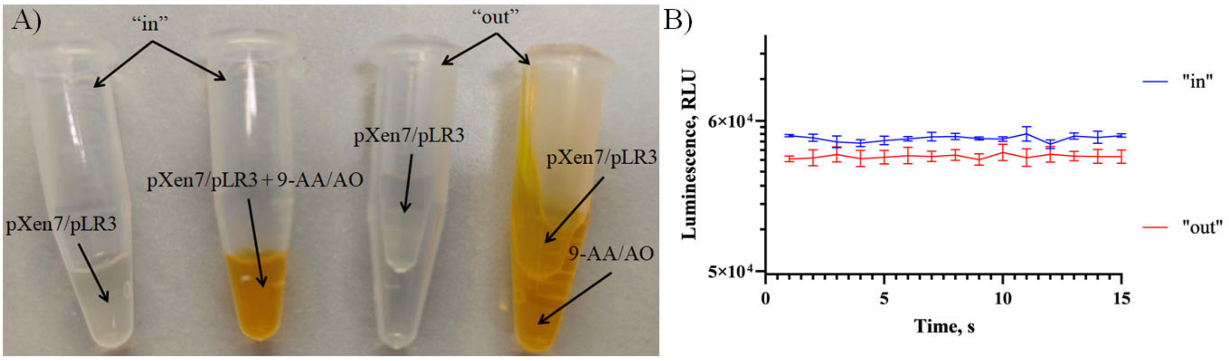

Figure S1. (**A**) Two sample preparation methods for *E. coli* pXen7 and *E. coli* pLR3. Cell suspensions are mixed with 9-AA or AO in a 1.5 mL test tube, which corresponds to the "in" variant (left). Cell suspensions in a small 0.5 mL test tube, which is inserted into a 1.5 mL test tube supplemented with 9-AA or AO (right) according to the "out" variant. Examples are shown before and after the addition of AO. (**B**) Luminescence measurements for 15 s of *E. coli* pXen7 suspensions prepared according to the "in" and "out" methods, but without the addition of the test substances. The difference in luminescence between the two samples is 1%.

The percentage luminescence values of the *E. coli* pXen7 and *E. coli* pLR3 cell suspensions are given in Figure S2 according to the "in" and "out" variants. The data were obtained as described in the main text of the article for 15 s before and 15 s after the addition of AO, depending on the concentration of the substance. Figure S3 represents the same, but 9-AA is used instead of AO. The luminescence values at the moment before substance addition are taken as 100%. The percentages of luminescence correspond to the data in Table 4 of the main text and are given on the ordinate axis near the corresponding dotted lines. The standard deviation is calculated based on three replicates.

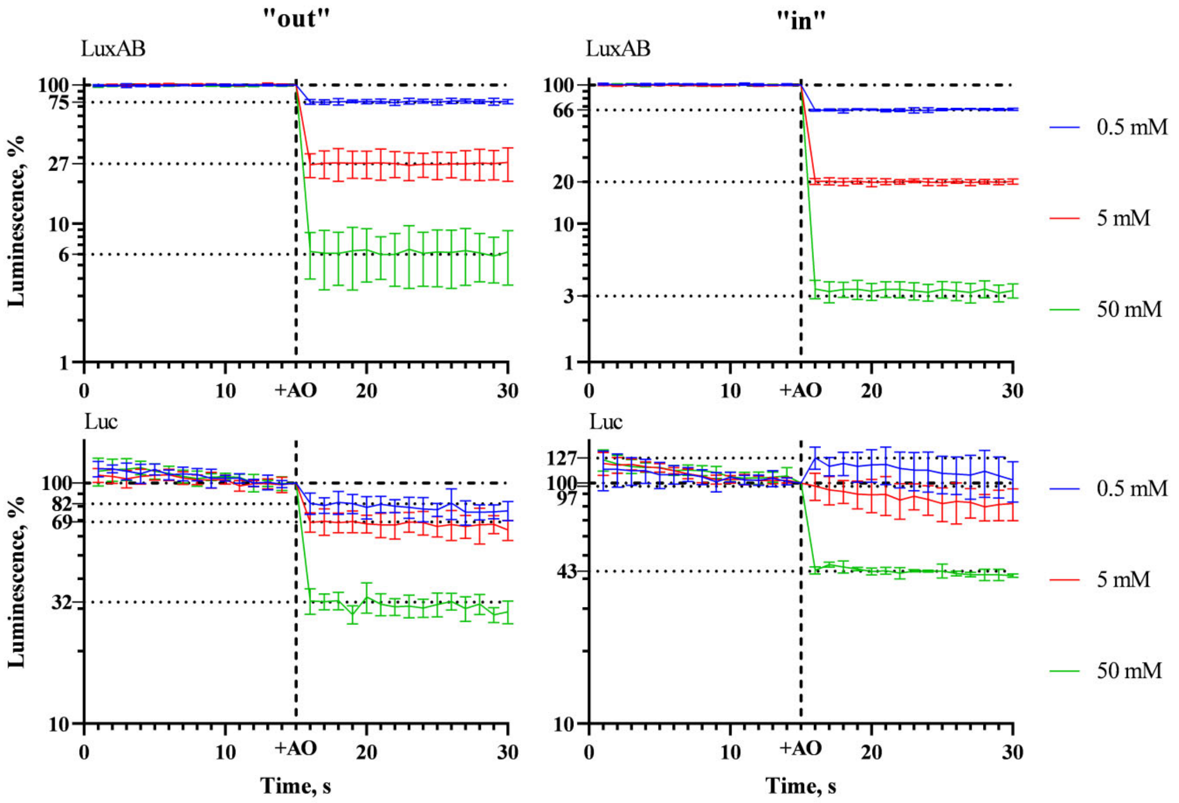

Figure S2. Percentage luminescence values obtained according to the method described in the main text of the article for *E. coli pXen7* (top, LuxAB) and *E. coli* pLR3 (bottom, Luc) according to the “out” (left) and “in” (right) variants, depending on the final AO concentrations: 0.5 (blue), 5 (red), and 50 (green) mM. The data were obtained for 15 s before (dashed line) and 15 s after AO supplementation. The luminescence values at the moment before AO addition are taken as 100% (dash-dotted line). The proportions of luminescence immediately after AO addition are indicated on the graphs by dotted lines; the corresponding numerical values are located near their intersection with the ordinate axis.

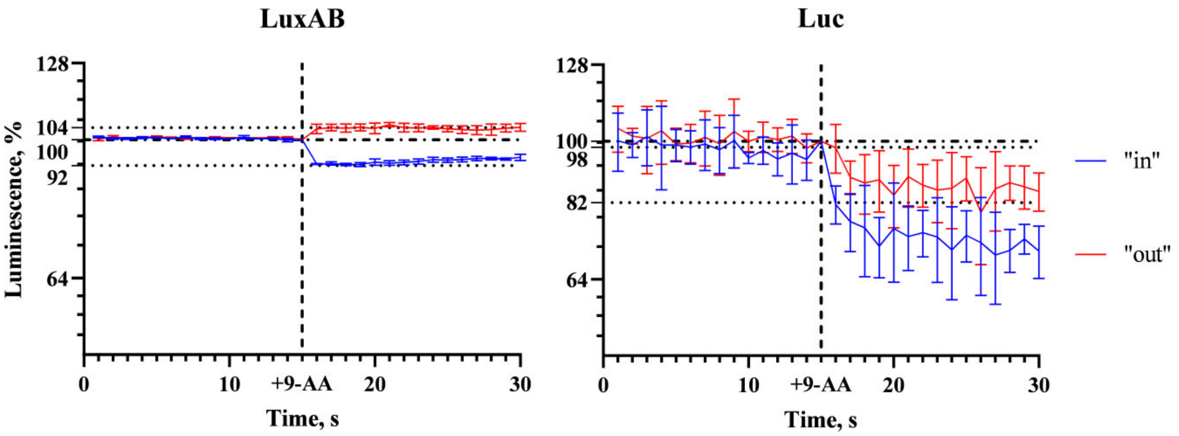

Figure S3. Percentage luminescence values obtained according to the method described in the main text of the article for *E. coli pXen7* (left, LuxAB) and *E. coli* pLR3 (right, Luc) according to the “out” (red) and “in” (blue) variants at a 9-AA concentration of 50 mM. The data were obtained for 15 s before (dashed line) and 15 s after 9--AA supplementation. The luminescence values at the moment before 9-AA addition are taken as 100% (dash-dotted line). The proportions of luminescence

immediately after 9-AA addition are indicated on the graphs by dotted lines; the corresponding numerical values are located near their intersection with the ordinate axis.

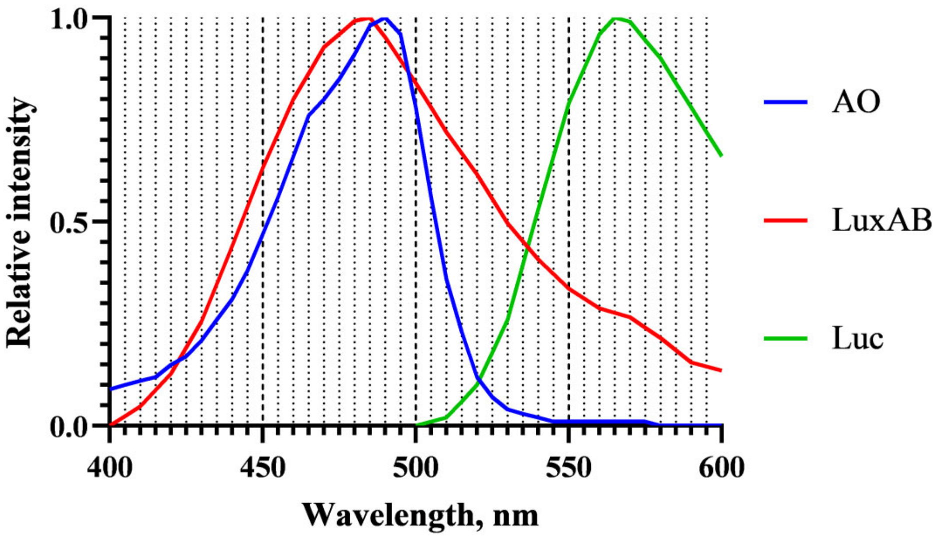

Figure S4. Normalized spectra: AO absorption [1], bacterial luciferase (LuxAB) emission [2], and firefly luciferase (Luc) emission [3].

References

1. Absorption [Acridine Orange] | AAT Bioquest Available online: https://www.aatbio.com/absorbance-uv-visible-spectrum-graph-viewer/acridine_orange (accessed on 30 October 2025).
2. Colepicolo, P.; Cho, K.W.; Poinar, G.O.; Hastings, J.W. Growth and Luminescence of the Bacterium Xenorhabdus Luminescens from a Human Wound. Applied and Environmental Microbiology 1989, 55, 2601–2606, doi:10.1128/aem.55.10.2601-2606.1989.
3. Koksharov, M.I.; Ugarova, N.N. Triple Substitution G216N/A217L/S398M Leads to the Active and Thermostable Luciola Mingrelica Firefly Luciferase. Photochemical and Photobiological Sciences 2011, 10, 931–938, doi:10.1039/C0PP00318B/METRICS.
